# Quality of life and impact of bile reflux after retro colic retro gastric gastrojejunostomy in Whipple surgery

**DOI:** 10.1186/s12876-017-0573-1

**Published:** 2017-01-21

**Authors:** Rohan Chaminda Siriwardana, Rajapaksha Walimuni Mudiyanselage Anuradha Lokubandara, Shivanthi Janaki De Silva Hewavisenthi, Saumya Kumuduni Liyanage, Dona Subani Priyangika Jayatunge, Chandika Anuradha Habarakada Liyanage

**Affiliations:** 1 Department of Surgery, Faculty of Medicine, University of Kelaniya, Ragama, Sri Lanka; 2 Department of Pathology, Faculty of Medicine, University of Kelaniya, Ragama, Sri Lanka

**Keywords:** Whipple surgery, Bile reflux, Quality of life

## Abstract

**Background:**

Delayed gastric emptying and bile reflux are common concerns in long-term survivors after Whipple surgery. The study was designed to assess modified retro colic retro gastric gastrojejunostomy in reducing macro and microscopic bile reflux and impact on dyspepsia related quality of life in long-term survivors.

**Methods:**

Out of 43 patients operated, 23 long-term survivors were included. All underwent gastroscopy and bile reflux was grouped as normal, yellowish bile lakes and presence of greenish bile lakes. Six standard gastric biopsies were taken. Microscopic bile reflux index (BRI) was calculated and a score more than 14 was considered significant. Validated Nepean dyspepsia index-short form (NDI-SF) was used to assess the severity of dyspepsia-related quality of life and compared with age and gender-matched control.

**Results:**

The median age was 48 (21–70) years. Median survival of the group was 37 months (6–40). Endoscopically, 20/23 (87%) had macroscopic bile reflux (74% yellowish bile lakes, 13% greenish bile lakes). None had stomal ulcers or macroscopic inflammation. Mean bile reflux index score was 9.7 (range 1.77–34). Mean NDI–SF score of Whipple group was 23.1 (SD 8.88). In controls, mean score was 19.9 (SD 8.23), showing no significant difference (*p* = 0.245).

**Conclusions:**

Though there was macroscopic bile reflux, clinical symptoms and microscopic changes were minimal. The modified technique had good long-term results.

## Background

Whipple surgery was once a procedure associated with high morbidity and mortality rates. However over the years, perioperative outcome has improved. Currently it is a safe surgery with low morbidity and mortality [1, 2]. Now the focus has shifted from improving perioperative outcome to improving long-term survival and quality of life [3, 4] . Delayed gastric emptying and bile reflux gastritis are the main concerns of long-term survivors [3]. Many surgical techniques are being used by different centers to overcome this. Pylorus preservation, the use of separate loop to drain bile and use of retro colic loop are some of the adaptations used [5–7]. Khan et.al described a novel technique in performing the gastrojejunostomy and reported superior early results with the technique [8]. During this reconstruction, posterior gastrojejunostomy is pulled in to the infra colic compartment and anchored to the transverse mesocolon. This helps the anastomosis to retain in a dependent anatomical position. However long-term outcome is not reported. This study looks at intermediate results of patients who underwent retro colic retro gastric gastrojejunostomy with a mesocolic stich.

## Methods

**Fig. 1:**
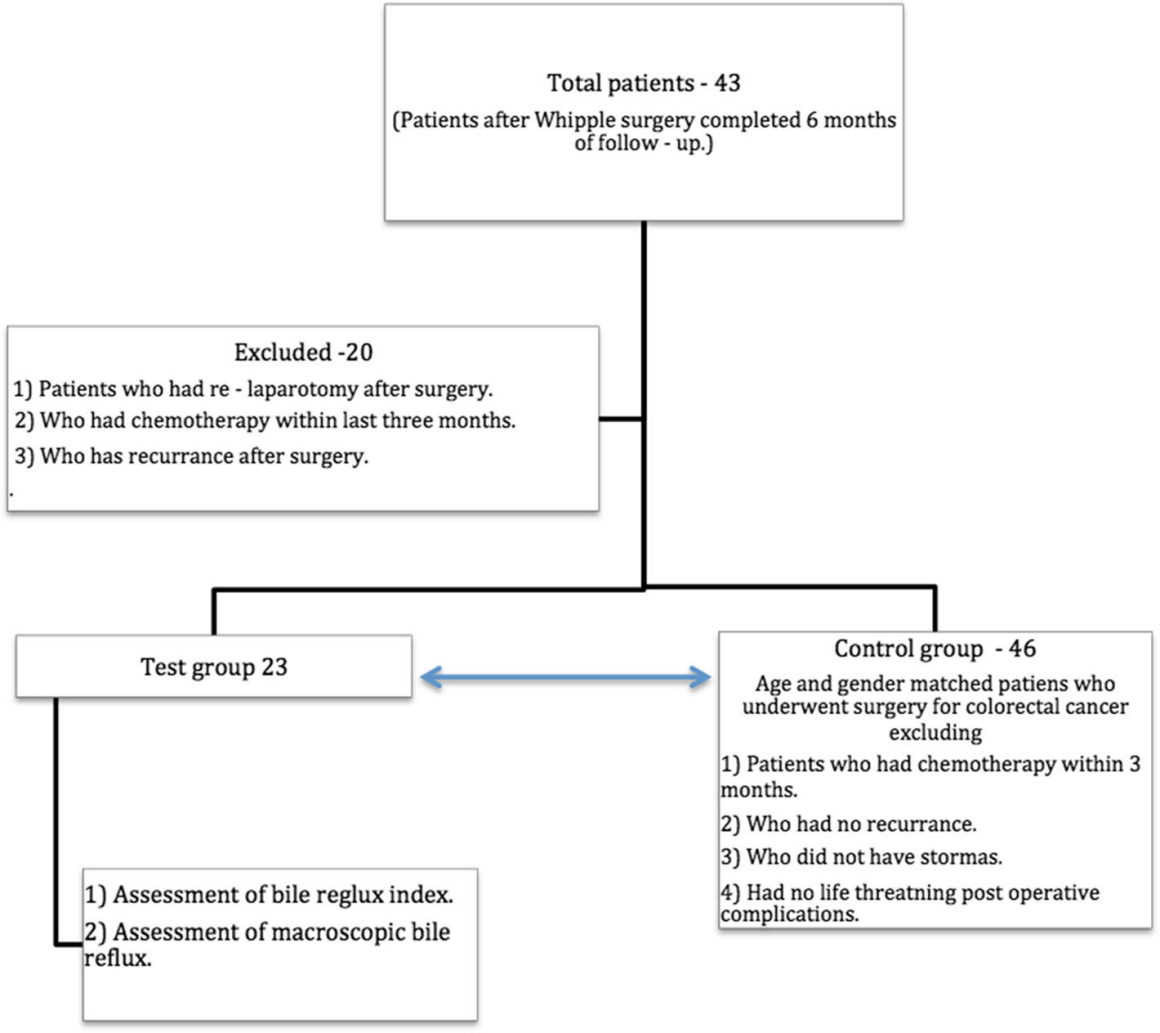
Selection of patients and controls

Forty three patients who underwent Whipple surgery from January 2012 for malignant disease were included in the study. Patients with recurrence of primary malignancy, those who had immediate post-operative complications that needed relaporotomy, those who underwent chemotherapy within the last 6 months, and those who did not consent were excluded from the study. There were 23 patients who fulfilled the inclusion criteria (Fig. 1). There were no pancreatic leaks in the group. All surgeries were done by two hepatobiliary surgeons who worked in parallel. A standard technique was used for Whipple surgery in all cases. The common bile duct was divided at the level of the cystic duct. All the nodes to the right of the common hepatic artery were taken as specimen. Pancreatic division was done at the level of the neck. The uncinate process was completely removed clearing all the tissues to the right of the superior mesenteric artery. The stomach was divided at the level of the incisura. After the dissection, reconstruction was done using a single Roux loop taken through a window in the transverse mesocolon. The pancreas was anastomosed using ‘end to side two layer’ technique. This was followed by ‘end to side biliary reconstruction’. A 60 cm gap was left between hepaticojejunostomy and gastrojejunostomy. A separate mesenteric window was made in the left edge of the transverse mesocolon and the small bowel was taken to the supracolic compartment through this window. A two layer longitudinal 4 cm hand-sewn gastrojejunostomy was made in the posterior surface of the stomach close to the distal end of the gastric stump. The anastomosed distal stomach was then pulled down in to the infracolic compartment. The gastric stump was anchored to the mesenteric window to prevent retraction. All 23 patients were being followed up at the hepatobiliary clinic at regular intervals.

The patients were evaluated at 6 months after the surgery. The patients who fulfilled the inclusion criteria underwent upper gastric endoscopy. Two observers documented the macroscopic appearance of the gastric mucosa. Sample color photographs were used as a guide to categorize the macroscopic appearance. Macroscopic appearance was categorized as normal, presence of yellowish bile lakes, presence of greenish bile lakes, presence of gastric inflammation and presence of stromal ulcers. Six biopsies were taken from six standard sites to represent the stomach. Biopsy specimens were stained with Hematoxylin and Eosin. Giemsa stain was done to detect Helicobacter pylori. Two pathologists evaluated all specimens separately. Discrepancies in scores were resolved through discussion until both pathologists agreed on common scores. Bile reflux was reported using a previously published bile reflux index (BRI). The index was calculated using the formula: 7 x oedema of lamina propria + 3 x intestinal metaplasia + 4 x chronic inflammation—6 x *H.pylori*. An index over 14 was considered significant.

Patient’s quality of life was assessed using Nepean dyspepsia short form quality of life (NDI-SF) questionnaire [9]. The questionnaire was translated to native language and was validated before using. Each patient was matched with two controls according to the age and gender. Controls were selected from a cohort of patients who had undergone lower abdominal surgeries for colorectal cancer. Patients who had recurrent disease, patients who had stomas, patients with bowel related complications or patients who were on chemotherapy within last 3 months were excluded. The NDI-SF score was compared between the two groups.

The study was approved by the Ethical Review Committee of the Faculty of Medicine, University of Kelaniya. All data were analyzed using SPSS 20. A *p* value of less than 0.05 was considered significant.

## Results

The median survival of the group was 37 (6–40) months. 10 (45%) were males. The median age was 48 (21–70) years. Median body mass index was 20.32 kg/m^2^ (15.03–30.07). 16 (69%) of them had diabetes. There were 10 (43%) patients with pancreatic cancers, 2 (8.6%) with bile duct cancers, 2 (8.6%) with neuro-endocrine tumours, and 9 (40%) with periampullary cancers. None of the patients in the group had early delayed gastric emptying. 6 (26%) Patients had adjuvant chemotherapy.

During endoscopy, 17 (74%) had yellowish bile lakes and 3 (13%) had greenish bile lakes. None of them had macroscopic gastric inflammation or stromal ulcers. In the microscopic assessment, the median BRI in the group was 9.7 (range 1.77–34). Only 5 patients had BRI over 14. *Helicobacor pylori* was not positive in any of the biopsies taken.

The quality of life was assessed in 10 areas. The overall score in the control was 19.9 (SD 8.23) while it was 23.1 (SD 8.88) in the test group indicating no significant difference (*p* = 0.245). The scores were comparable in the individual areas of NDI—SF (Table 1).

**Table 1:** Nepean dyspepsia index in the test and the control groups

	Study group mean (SD)	Control group mean (SD)	*P* value
General emotional well-being been disturbed by stomach problems	2.10 (1.07)	2.45 (1.39)	0.615
Irritable, tense or frustrated because of stomach problems	2.30 (1.13)	1.95 (1.10)	0.530
Ability to engage in things usually do for fun been disturbed by stomach problems	2.55 (1.47)	2.05 (1.32)	0.772
Enjoyment of things done usually for fun been disturbed by stomach problems	2.10 (1.33)	2.75 (1.48)	0.396
Ability to eat or drink been disturbed by stomach problems	2.15 (1.50)	2.10 (1.25)	0.726
Enjoyment of eating and/or drinking been disturbed by stomach problems	2.10 (1.25)	2.15 (1.18)	0.687
Feeling that they will always have stomach problems	2.45 (1.43)	2.00 (0.92)	0.353
Feeling of stomach problems might be due to a very serious illness.	2.05 (1.43)	1.75 (1.07)	0.289
Ability to work or study been disturbed by stomach problems	2.50 (1.70)	1.40 (1.23)	0.222
Enjoyment of work or study been disturbed by stomach problems	2.50 (1.70)	1.40 (1.14)	0.132
Total score	23.10 (8.88)	19.90 (8.23)	0.245

## Discussion

In this cohort who had intermediate survival after Whipple surgery, bile was commonly noted in the distal part of the gastric stump. However biopsies did not show significant microscopic changes of bile reflux. Their quality of life was also comparable to the control. Maintaining the gastric stump in near-anatomical position, preventing stump retraction and angulation are considered important causes for good functional results after gastrectomy [6]. In the technique we use, the stomach is anchored to the root of the mesentery that helps to maintain its position. Furthermore there may be a mechanical advantage of keeping the gastric anastomosis in the infracolic compartment that facilitate the rapid emptying of the bile that enters in to the stomach.

Bile reflux gastritis is a well known histological entity characterized by foveolar hyperplacia, mucosal oedema, congestion and presence of acute and chronic inflammation [10]. Persistent bile reflux into the gastric remnant is known to cause significant clinical symptoms, structural changes and gastric carcinoma [10, 11]. However data looking specifically at bile reflux after Whipple surgery is limited in literature. Most data are from studies that used gastrectomy for distal gastric or duodenal cancer. In these studies, Roux en Y reconstruction was used to overcome bile reflux after distal gastrectomy [12, 13]. However, mobilizing a separate limb for biliary diversion adds more surgical time in addition to inherent problems of the Roux loop [14]. Another technique used is Braun enterostomy which has shown to reduce the incidence of bile reflux and delayed gastric emptying [15]. Additional jejunojejunostomy prolongs the surgical time and can lead to other anastomosis related complications. In comparison to both these techniques, single loop reconstruction that we used is simple and is easy to perform.

Macroscopic bile reflux was noted in 87% of our cases. Bile pooled mainly in the distal stomach. Previous data from Fukuhara et.al [15] and others [16, 17] had shown a significant relationship between mucosal inflammation and presence of *Helicobacter pylori* infection. None of our patients had *Helicobacter pylori* present in the gastric mucosa. This could be an additional factor for not having significant mucosal inflammation. The median time for our evaluation since the surgery was 37 months. In a previous study, patients presenting with symptoms after gastrostomy showed bile reflux gastritis (BRG) in 60% of the cases after 14 years of surgery [18]. However other studies have shown mucosal changes much earlier [19]. Therefore median follow-up of 37 months in our cohort seems to be adequate to develop mucosal changes.

Delayed gastric emptying (DGE) is a well known complication after Whipple surgery. Incidence of early DGE is recorded to be around 20–30%. Its long-term impact and incidence of persistent symptoms is rarely discussed. Delayed gastric emptying is reported in around 10–20% of patients at 3 years after Whipple surgery [20]. In another study using standard reconstruction showed a similar incidence of DGE assessed with C13 acetate breath test at 24 months after surgery [21]. However gastric emptying studies need to be interpreted carefully in these patients who had lost a significant part of the stomach. In our study, gastric emptying time was not assessed. We feel that results of emptying studies have a little value in the absence of significant mucosal changes and symptoms.

One of the main drawbacks in the study is that it evaluates a smaller cohort of patients. Since this is the standard technique used in the center for reconstruction, we were not able to compare with a cohort reconstructed with an alternate technique. Since bile reflux index is a standard method in assessing bile reflux, we feel that having a lower BRI score without comparison is acceptable in arriving at our conclusion. However quality of life is best compared with a cohort having alternative reconstruction technique. To overcome this we selected a group of patients who had colonic surgeries unlikely to have upper gastric symptoms.

## Conclutions

Patients with retro colic retro gastric gastrojejunostomy have good quality of life and minimal gastric mucosal changes. The technique seems to give good long-term results after Whipple surgery. This technique needs to be evaluated in a larger cohort.

## Abbreviations

BRI: Bile reflux Index
NDI-SF: Nepean dyspepsia index-short form
